# Targeting the YAP/TAZ mechanotransducers in solid tumour therapeutics

**DOI:** 10.1111/jcmm.17794

**Published:** 2023-05-25

**Authors:** Antonios N. Gargalionis, Kostas A. Papavassiliou, Athanasios G. Papavassiliou

**Affiliations:** ^1^ Department of Biopathology, ‘Eginition’ Hospital, Medical School National and Kapodistrian University of Athens Athens Greece; ^2^ First University Department of Respiratory Medicine, ‘Sotiria’ Hospital, Medical School National and Kapodistrian University of Athens Athens Greece; ^3^ Department of Biological Chemistry, Medical School National and Kapodistrian University of Athens Athens Greece

**Keywords:** ECM stiffness, mechanotransducer, solid tumour, therapeutic targeting, YAP/TAZ

Extracellular matrix (ECM) interacts with solid tumour cells and cells from the tumour microenvironment (ΤΜΕ). Components of the ECM and various types of mechanical forces which are generated from ECM stiffening modulate properties of solid tumour cells, thereby affecting tumour proliferation, migration, angiogenesis, immune evasion, stemness and resistance to treatment. Clarification of the role of ECM‐associated protein molecules during solid tumour progression offers new therapeutic strategies aiming at overcoming ECM‐mediated oncogenic signalling and drug resistance.^1^, ^2^

A well‐investigated signalling pathway linked to alterations of ECM rigidity is the Hippo signal transduction pathway. It is an evolutionary conserved kinase cassette that controls cell proliferation, apoptosis, self‐renewal of stem cells and organ size, and is regulated by mechanical cues, the status of cellular energy and multiple receptor signalling.^3^ Yes‐associated protein (YAP) and transcriptional coactivator with PDZ‐binding motif (TAZ) are key transcriptional regulators and the end nuclear effectors of the Hippo signal transduction cascade. YAP and TAZ are transcription coactivators and function by tethering to the transcriptional enhanced associate domain (TEAD) transcription factor (there are four TEAD proteins (TEAD1‐4) in the human genome).^3^ Following activation of the Hippo pathway, the unphosphorylated Merlin protein phosphorylates/potentiates STE20‐like protein kinase 1/2 (MST1/2)/large tumour suppressor 1/2 (LATS1/2) proteins to turn off YAP/TAZ by multiple‐site phosphorylation. YAP/TAZ inactivation leads to YAP/TAZ cytoplasmic retention. Conversely, inactivation of the Hippo pathway leads to YAP/TAZ nuclear accumulation and target gene transcription.^3^

Changes in ECM stiffness elicit mechano‐stimulated tumorigenic mechanisms through activation of YAP/TAZ (Figure 1). Activated YAP/TAZ induce the expression of genes that promote cell proliferation.^3^ For example, in oral squamous cell carcinoma, YAP potentiation triggers transcriptional activation of piezo type mechanosensitive ion channel component 1 (Piezo1), a mechano‐induced Ca^2+^ channel, and this evokes tumour cell proliferation.^4^ YAP activation also promotes cell migration under stiffer ECM microenvironment, for example hepatocellular carcinoma (HCC) cells present increased migration in vitro when cultured under stiffer extracellular conditions. Mechanistically, stiffer ECM ignites c‐Jun N‐terminal kinase (JNK) and p38 mitogen‐activated protein kinases (MAPK) signalling leading to induction of YAP and its translocation to the nucleus. Subsequently, YAP transcriptionally mediates enhancement of aerobic glycolysis and migration of HCC cells.^5^ An alternative route involves upregulation of C‐X‐C motif chemokine receptor 4 (CXCR4), a known booster of HCC, by ECM stiffness. CXCR4 downregulates ubiquitin domain‐containing protein 1 (UBTD1), thereby decreasing poly‐ubiquitination and proteasome‐dependent degradation of YAP. Sustainable YAP expression transcriptionally activates tumour‐promoting genes.^6^

**FIGURE 1:**
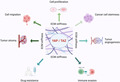
Regulation of tumour cell properties by YAP/TAZ. YAP/TAZ transcriptional coactivators mediate mechanical signals induced by ECM stiffening and drive cancer cell proliferation, migration, stemness, angiogenesis, interactions with tumour stroma, immune evasion and drug resistance. CD8, cluster of differentiation 8; ECM, extracellular matrix; MHC, major histocompatibility complex; TAZ, transcriptional coactivator with PDZ‐binding motif; TCR, T‐cell receptor; YAP, yes‐associated protein. This figure was created based on the tools provided by Biorender.com (accessed on 28/04/2023).

It has been documented that YAP/TAZ activation mediates resistance to pharmacological intervention as a result of changes in ECM stiffness.^7^ Biochemical and mechanical stimuli in the tumour stroma due to alterations of ECM stiffness participate in metastases and drug resistance.^8^ For instance, actin remodelling activates YAP/TAZ in melanoma cells and this event fosters resistance to serine/threonine‐protein kinase B‐Raf inhibitors (BRAFi).^9^ Furthermore, YAP/TAZ regulate programmed death‐ligand 1 (PD‐L1) expression and YAP specifically mediates downregulation of the antitumour immune response in BRAFi‐resistant melanoma cells in a PD‐L1‐dependent manner.^10^ Alterations in ECM rigidity can also cause resistance to the human epidermal growth factor receptor 2 (HER2) inhibitors (e.g. lapatinib) through YAP/TAZ in breast cancer.^11^ Data from breast cancer cells reveal that intermediate‐stiffness substrates are correlated with decreased uptake, increased efflux and resistance to doxorubicin, compared to high‐ and low‐stiffness substrates. Doxorubicin resistance is associated with higher expression levels of the mechanosensory protein integrin‐linked kinase (ILK) and activation of YAP. ILK can regulate YAP expression and activity in an intermediate ECM stiffness status and confer drug resistance to breast cancer cells.^12^ In a similar vein, chemoresistance in ovarian cancer is driven by chemotherapy‐induced alterations in ECM stiffness and composition. ECM microenvironment evolves during platinum‐based chemotherapy (cisplatin, carboplatin), as demonstrated by distinct expression profiles of core ECM‐related genes in pre‐ and post‐chemotherapy samples. Stiffer ECM and platinum treatment enhances spreading, focal adhesion formation and nuclear translocation of YAP/TAZ in high‐grade serous carcinoma (HGSC) ovarian cells. Moreover, ECM stiffness functionally contributes to resistance of ovarian cancer cells to platinum‐triggered apoptosis via focal adhesion kinase (FAK) and YAP signalling. Platinum‐mediated resistance is attributed to activation of specific ECM proteins, such as vitronectin (VTN), fibronectin (FBN) and collagen VI (ColVI).^8^ On the contrary, chemotherapy resistance to cisplatin and doxorubicin in HCC is attributed to specific TAZ upregulation. TAZ can transcriptionally activate interleukin 8 (IL‐8) to confer resistance; therefore, TAZ‐IL‐8 is a reasonable axis for therapeutic targeting.^13^ YAP/TAZ upregulation, in synergy with activating transcription factor 4 (ATF4), are also responsible for resistance to the multikinase inhibitor sorafenib in HCC.^14^ In experiments where hepatocellular tumour spheroids were employed, YAP/TAZ activation occurs in tumour and stromal cells of HCC.^15^ In a patient‐derived hepatocellular tumour spheroid model, YAP inhibition can sensitize HCC cells with high YAP/TAZ expression to kinase inhibition by sorafenib.^16^ It should also be taken into consideration that although YAP functions as an oncoprotein that confers drug resistance, recent findings suggest that YAP may also act as a tumour suppressor. Androgen receptor (AR) interacts with TEAD to promote tumour growth in prostate cancer cells. YAP upregulation competes with AR for TEAD binding to suppress TEAD‐mediated AR signalling and tumour progression in AR‐positive prostate cancer. YAP can also impede anti‐hormone therapy resistance caused by AR variants. This indicates that pharmacological targeting of the Hippo pathway may be effective to overcome resistance to anti‐androgen treatment in prostate cancer.^17^

As most solid tumour cells depend on their mechanical properties to promote their malignant features, small‐molecule compounds (derived from available banks or chemically synthesized) targeting YAP/TAZ, either directly or by disrupting vital interactions with transcription factors/complexes, have been developed and have already progressed to clinical trials with promising results.^2^, ^7^ A rational design of YAP/TAZ targeting involves hindering of the intermolecular interaction between TEAD and its coactivators YAP/TAZ. This seems an attractive therapeutic strategy to treat solid tumours with a dysregulated Hippo pathway.^18^ However, it has been difficult to locate suitable hotspot binding sites conducive to abrogate YAP/TAZ‐TEAD interactions. YAP binds to the α‐helix and Ω‐loop distinct pockets of TEAD, which have been shown to be crucial drug‐targeting inhibitory sites of the YAP‐TEAD complex.^18^ The first small non‐peptide molecules to disrupt the YAP‐TEAD interaction that have been identified bind competitively to the specific Ω‐loop pocket of TEAD, as they display the same level of affinity for TEAD as YAP does.^18^ A subsequent study offered further optimization of such Ω‐loop‐targeting class of low‐molecular mass compounds and proved to be efficient in tumour‐bearing mice following oral administration.^19^ Concerning peptide inhibitors, albeit more progress has been made in targeting the Ω‐loop region, a peptide inhibitor with high affinity to the α‐helix pocket was recently biochemically and structurally characterized.^20^ In gastrointestinal cancers, two peptide segments, which represent an α‐helix and an Ω‐loop, have been isolated from binding sites of YAP/TAZ with TEAD and are promising candidates for Hippo pathway targeting. Nevertheless, these self‐inhibitory peptides are easily dissociated when they are not parts of the full‐length proteins; hence, they are not able to competitively annul binding of TEAD to YAP/TAZ. To this end, a new rational design methodology was recently proposed that employs a chemical modification, termed hydrocarbon stapling technique, to retain the conformation of the α‐helix peptide. This approach can improve peptide binding to TEAD and interfere effectively with coactivators‐transcription factor complex formation.^21^

YAP is also implicated in the angiogenic process of diverse solid tumours and small‐molecule inhibitors targeting that aspect of YAP function have been developed. Verteporfin (VP) is a low‐molecular mass compound that suppresses YAP signalling by hampering YAP/TEAD interactions and can restrain proliferation, migration, tube formation and facilitate apoptosis of human umbilical vein endothelial cells (HUVECs).^22^ It causes reduction of massive vessel branches production, reduction of the number of blood vessels in vivo and downregulation of the expression of angiogenesis‐engaged genes. Thus, VP has been shown to potently attenuate malignant progression of oesophageal squamous cell carcinoma (ESCC) cells via inhibition of tumour angiogenesis.^22^ Additionally, targeting angiogenesis can re‐sensitize ESCC cells to treatment with paclitaxel.^22^ VP targeting of the YAP/TAZ pathway is also effective to overcome drug resistance in endometrial carcinoma (EC). YAP/TAZ mediates resistance to progestin through the phosphoinositide 3‐kinase (PI3K)/Akt pathway, and VP sensitizes EC cells to progestin both in vitro and in vivo.^23^ It also demonstrates an additive antitumor effect when combined with the histone demethylase lysine‐specific demethylase 1 (LSD1) inhibitor SP2509 in vitro and in vivo in oral squamous cell carcinoma cells.^24^

There are also selective inhibitors of TEAD. K‐975 is a newly identified highly selective TEAD inhibitor that impairs YAP/TAZ‐TEAD protein–protein interactions. It exerts antitumor effects by suppressing YAP/TAZ‐TEAD signalling in pleural mesothelioma.^25^ Another (oral) TEAD inhibitor, IK‐930, is currently in phase 1 clinical trial evaluation in patients with advanced solid tumours bearing or not gene alterations in the Hippo pathway (NCT05228015). Data regarding the TEAD palmitoylation inhibitor MGH‐CP1 imply that TEAD inhibition alone is not enough to cause cancer‐cell death, because it transcriptionally activates an alternative pathway of cell survival through the sex‐determining region Y‐related high‐mobility‐group box transcription factor 4 (SOX4)/PI3K/Akt axis. Dual targeting and inhibition of TEAD and Akt represents an efficient treatment option.^26^ Lastly, IAG933, a potent and direct small‐molecule disruptor of the YAP‐TEAD protein–protein interaction, is presently in clinical trial evaluation in patients with mesothelioma, neurofibromatosis 2 (NF2, also known as Merlin)/LATS1/2‐mutated tumours and tumours with functional YAP/TAZ fusions (i.e. YAP/TAZ hybrids that hyperactivate a TEAD‐based transcriptome) (NCT04857372), but its exact mechanism of action has not yet been disclosed.^27^, ^28^

Recent findings further implicate YAP/TAZ in signalling circuitries of the ΤΜΕ as candidate targets to improve chemotherapy efficacy. Nonetheless, as shown in breast tumours, stage‐specific YAP/TAZ expression profiles and/or crosstalk with co‐operating signalling molecules should also be taken into account in predicting response to combinatorial YAP/TAZ/TEAD‐targeted treatment approaches.^29^, ^30^

Conclusively, accumulating evidence suggests that anomalous YAP/TAZ signalling contributes significantly to solid tumour development and progression and is a critical factor of intrinsic and acquired resistance to a variety of targeted regimens and chemotherapies. Further research is required to elucidate the underpinning mechanisms of YAP/TAZ operational aberrations in order to formulate more efficacious treatments for a broad spectrum of solid tumours and their clinical complications.

## AUTHOR CONTRIBUTIONS

**Antonios N. Gargalionis:** Conceptualization (equal); data curation (equal); writing – original draft (lead). **Kostas A. Papavassiliou:** Conceptualization (equal); data curation (equal); writing – original draft (equal). **Athanasios G. Papavassiliou:** Conceptualization (lead); data curation (lead); supervision (lead); writing – review and editing (lead).

## CONFLICT OF INTEREST STATEMENT

The authors declare no competing financial interests.

## Data Availability

Data sharing is not applicable—no new data generated.

## References

[1] Hayward MK , Muncie JM , Weaver VM . Tissue mechanics in stem cell fate, development, and cancer. Dev Cell. 2021;56(13):1833‐1847. 34107299 10.1016/j.devcel.2021.05.011 PMC9056158

[2] Jiang Y , Zhang H , Wang J , Liu Y , Luo T , Hua H . Targeting extracellular matrix stiffness and mechanotransducers to improve cancer therapy. J Hematol Oncol. 2022;15(1):34. 35331296 10.1186/s13045-022-01252-0 PMC8943941

[3] Li FL , Guan KL . The two sides of hippo pathway in cancer. Semin Cancer Biol. 2022;85:33‐42. 34265423 10.1016/j.semcancer.2021.07.006

[4] Hasegawa K , Fujii S , Matsumoto S , Tajiri Y , Kikuchi A , Kiyoshima T . YAP signaling induces PIEZO1 to promote oral squamous cell carcinoma cell proliferation. J Pathol. 2021;253(1):80‐93. 32985688 10.1002/path.5553

[5] Liu QP , Luo Q , Deng B , Ju Y , Song GB . Stiffer matrix accelerates migration of hepatocellular carcinoma cells through enhanced aerobic glycolysis via the MAPK‐YAP signaling. Cancers (Basel). 2020;12(2):490. 32093118 10.3390/cancers12020490 PMC7072284

[6] Yang N , Chen T , Wang L , et al. CXCR4 mediates matrix stiffness‐induced downregulation of UBTD1 driving hepatocellular carcinoma progression via YAP signaling pathway. Theranostics. 2020;10(13):5790‐5801. 32483419 10.7150/thno.44789 PMC7255012

[7] Kim HB , Myung SJ . Clinical implications of the hippo‐YAP pathway in multiple cancer contexts. BMB Rep. 2018;51(3):119‐125. 29366445 10.5483/BMBRep.2018.51.3.018 PMC5882218

[8] Pietila EA , Gonzalez‐Molina J , Moyano‐Galceran L , et al. Co‐evolution of matrisome and adaptive adhesion dynamics drives ovarian cancer chemoresistance. Nat Commun. 2021;12(1):3904. 34162871 10.1038/s41467-021-24009-8 PMC8222388

[9] Kim MH , Kim J , Hong H , et al. Actin remodeling confers BRAF inhibitor resistance to melanoma cells through YAP/TAZ activation. EMBO J. 2016;35(5):462‐478. 26668268 10.15252/embj.201592081 PMC4772854

[10] Kim MH , Kim CG , Kim SK , et al. YAP‐induced PD‐L1 expression drives immune evasion in BRAFi‐resistant melanoma. Cancer Immunol Res. 2018;6(3):255‐266. 29382670 10.1158/2326-6066.CIR-17-0320

[11] Lin CH , Pelissier FA , Zhang H , et al. Microenvironment rigidity modulates responses to the HER2 receptor tyrosine kinase inhibitor lapatinib via YAP and TAZ transcription factors. Mol Biol Cell. 2015;26(22):3946‐3953. 26337386 10.1091/mbc.E15-07-0456 PMC4710228

[12] Qin X , Lv X , Li P , et al. Matrix stiffness modulates ILK‐mediated YAP activation to control the drug resistance of breast cancer cells. Biochim Biophys Acta Mol basis Dis. 2020;1866(3):165625. 31785406 10.1016/j.bbadis.2019.165625

[13] Zhang H , Yu QL , Meng L , et al. TAZ‐regulated expression of IL‐8 is involved in chemoresistance of hepatocellular carcinoma cells. Arch Biochem Biophys. 2020;693:108571. 32898567 10.1016/j.abb.2020.108571

[14] Gao R , Kalathur RKR , Coto‐Llerena M , et al. YAP/TAZ and ATF4 drive resistance to Sorafenib in hepatocellular carcinoma by preventing ferroptosis. EMBO Mol Med. 2021;13(12):e14351. 34664408 10.15252/emmm.202114351 PMC8649869

[15] Cho K , Ro SW , Lee HW , et al. YAP/TAZ suppress drug penetration into hepatocellular carcinoma through stromal activation. Hepatology. 2021;74(5):2605‐2621. 34101869 10.1002/hep.32000

[16] Han S , Lim JY , Cho K , et al. Anti‐cancer effects of YAP inhibitor (CA3) in combination with Sorafenib against hepatocellular carcinoma (HCC) in patient‐derived multicellular tumor spheroid models (MCTS). Cancers (Basel). 2022;14(11):2733. 35681712 10.3390/cancers14112733 PMC9179573

[17] Li X , Zhuo S , Cho YS , et al. YAP antagonizes TEAD‐mediated AR signaling and prostate cancer growth. EMBO J. 2023;42(4):e112184. 36588499 10.15252/embj.2022112184 PMC9929633

[18] Furet P , Bordas V , Le Douget M , et al. The first class of small molecules potently disrupting the YAP‐TEAD interaction by direct competition. ChemMedChem. 2022;17(19):e202200303. 35950546 10.1002/cmdc.202200303

[19] Sellner H , Chapeau E , Furet P , et al. Optimization of a class of Dihydrobenzofurane analogs toward orally efficacious YAP‐TEAD protein‐protein interaction inhibitors. ChemMedChem. 2023;e202300051. doi:10.1002/cmdc.202300051 Epub ahead of print. 36988034

[20] Mesrouze Y , Gubler H , Villard F , et al. Biochemical and structural characterization of a Peptidic inhibitor of the YAP:TEAD interaction that binds to the alpha‐helix pocket on TEAD. ACS Chem Biol. 2023;18(3):643‐651. 36825662 10.1021/acschembio.2c00936

[21] Gao S , Wang Y , Ji L . Rational design and chemical modification of TEAD coactivator peptides to target hippo signaling pathway against gastrointestinal cancers. J Recept Signal Transduct Res. 2021;41(4):408‐415. 32912021 10.1080/10799893.2020.1818093

[22] Wang XW , Zhao R , Yang ZY , et al. YAP inhibitor verteporfin suppresses tumor angiogenesis and overcomes chemoresistance in esophageal squamous cell carcinoma. J Cancer Res Clin Oncol. 2023. doi:10.1007/s00432-023-04722-1 Epub ahead of print. PMC11796746 37000262

[23] Wei L , Ma X , Hou Y , et al. Verteporfin reverses progestin resistance through YAP/TAZ‐PI3K‐Akt pathway in endometrial carcinoma. Cell Death Dis. 2023;9(1):30. 10.1038/s41420-023-01319-y PMC9873621 36693834

[24] Alhousami T , Diny M , Ali F , et al. Inhibition of LSD1 attenuates Oral cancer development and promotes therapeutic efficacy of immune checkpoint blockade and YAP/TAZ inhibition. Mol Cancer Res. 2022;20(5):712‐721. 35105672 10.1158/1541-7786.MCR-21-0310 PMC9081163

[25] Kaneda A , Seike T , Danjo T , et al. The novel potent TEAD inhibitor, K‐975, inhibits YAP1/TAZ‐TEAD protein‐protein interactions and exerts an anti‐tumor effect on malignant pleural mesothelioma. Am J Cancer Res. 2020;10(12):4399‐4415. 33415007 PMC7783735

[26] Sun Y , Hu L , Tao Z , et al. Pharmacological blockade of TEAD‐YAP reveals its therapeutic limitation in cancer cells. Nat Commun. 2022;13(1):6744. 36347861 10.1038/s41467-022-34559-0 PMC9643419

[27] Zhao B , Pobbati AV , Rubin BP , Stauffer S . Leveraging hot spots of TEAD‐Coregulator interactions in the Design of Direct Small Molecule Protein‐Protein Interaction Disruptors Targeting Hippo Pathway Signaling. Pharmaceuticals (Basel). 2023;16(4):583. 37111340 10.3390/ph16040583 PMC10146773

[28] Schmelzle T , Chapeau E , Bauer D , et al. IAG933, a selective and orally efficacious YAP1/WWTR1(TAZ)‐panTEAD protein‐protein interaction inhibitor with pre‐clinical activity in monotherapy and combinations. Cancer Res. 2023;83(8_Suppl):LB319.

[29] Karamouzis MV , Badra FA , Papavassiliou AG . Breast cancer: the upgraded role of HER‐3 and HER‐4. Int J Biochem Cell Biol. 2007;39(5):851‐856. 17254832 10.1016/j.biocel.2006.11.017

[30] Yuan JQ , Ding NH , Xiao Z . The hippo transducer YAP/TAZ as a biomarker of therapeutic response and prognosis in Trastuzumab‐based neoadjuvant therapy treated HER2‐positive breast cancer patients. Front Pharmacol. 2020;11:537265. 32973536 10.3389/fphar.2020.537265 PMC7481481

